# Representation of Cognitive Reappraisal Goals in Frontal Gamma Oscillations

**DOI:** 10.1371/journal.pone.0113375

**Published:** 2014-11-17

**Authors:** Jae-Hwan Kang, Ji Woon Jeong, Hyun Taek Kim, Sang Hee Kim, Sung-Phil Kim

**Affiliations:** 1 Department of Brain and Cognitive Engineering, Korea University, Seoul, Republic of Korea; 2 Department of Psychology, Korea University, Seoul, Republic of Korea; 3 Department of Human and Systems Engineering, Ulsan National Institute of Science and Technology, Ulsan, Republic of Korea; University of L'Aquila, Italy

## Abstract

Recently, numerous efforts have been made to understand the neural mechanisms underlying cognitive regulation of emotion, such as cognitive reappraisal. Many studies have reported that cognitive control of emotion induces increases in neural activity of the control system, including the prefrontal cortex and the dorsal anterior cingulate cortex, and increases or decreases (depending upon the regulation goal) in neural activity of the appraisal system, including the amygdala and the insula. It has been hypothesized that information about regulation goals needs to be processed through interactions between the control and appraisal systems in order to support cognitive reappraisal. However, how this information is represented in the dynamics of cortical activity remains largely unknown. To address this, we investigated temporal changes in gamma band activity (35–55 Hz) in human electroencephalograms during a cognitive reappraisal task that was comprised of three reappraisal goals: to decease, maintain, or increase emotional responses modulated by affect-laden pictures. We examined how the characteristics of gamma oscillations, such as spectral power and large-scale phase synchronization, represented cognitive reappraisal goals. We found that left frontal gamma power decreased, was sustained, or increased when the participants suppressed, maintained, or amplified their emotions, respectively. This change in left frontal gamma power appeared during an interval of 1926 to 2453 ms after stimulus onset. We also found that the number of phase-synchronized pairs of gamma oscillations over the entire brain increased when participants regulated their emotions compared to when they maintained their emotions. These results suggest that left frontal gamma power may reflect cortical representation of emotional states modulated by cognitive reappraisal goals and gamma phase synchronization across whole brain regions may reflect emotional regulatory efforts to achieve these goals. Our study may provide the basis for an electroencephalogram-based neurofeedback system for the cognitive regulation of emotion.

## Introduction

An individual's ability to regulate emotional responses to external stimuli or internal mental representations relates not only to that individual's mental health but also to many social problems [1]–[3]. Accordingly, cognitive regulation of emotion, which refers to cognitive processes involved in influencing the onset, offset, intensity, or quality of emotional responses [4], has emerged as an important topic in many disciplines (i.e., psychology, psychiatry, and social neuroscience). Emotion regulation processes can be categorized based on whether they occur before or after an emotion is generated: antecedent-focused emotion regulation is applied earlier to alter the trajectory of emotion responses before they arise, whereas response-focused emotion regulation is applied later to modulate the emotional response after the emotion is generated [5]. Our study focuses on cognitive reappraisal, one type of antecedent-focused emotion regulation strategy. Cognitive reappraisal is considered an effective emotion regulation strategy and has received relatively more scientific attention than others have because it generally requires fewer cognitive demands and induces no memory impairment [6], [7].

Over the past decades, many neuropsychological studies have investigated how emotion regulation strategies modulate neural responses to emotional events. To this end, they directly measured brain activity during emotion regulation using various methods, such as electroencephalogram (EEG), magnetoencephalogram (MEG), and functional magnetic resonance imaging (fMRI). Studies using EEG have shown a close relationship between the arousal level modulated by an emotional stimulus and the amplitude of the late positive potential (LPP), which arises approximately 300 to 400 ms after stimulus onset, and persists throughout the duration of stimulus presentation [8], [9]. Other studies have demonstrated the LPP as an indicator of emotional perception [8], [10], [11], and suggested that it is modulated by a network of cortical and subcortical regions related to visual and emotional information processing [12]. Emotion regulation decreased LPP amplitude when individuals downregulated their emotional response to negative stimuli [13], [14].

Ochsner and colleagues proposed a functional architecture underlying cognitive regulation of emotion that consists of both a voluntary top-down cognitive control system implemented in the prefrontal cortex (PFC) and the dorsal anterior cingulate cortex (dACC), and an automatic bottom-up emotional appraisal system implemented in subcortical structures, including the amygdala and the insula [15]. Many fMRI studies have indicated that interactions between the control system and the appraisal system play a key role in cognitive control of emotion [16]–[20]. These studies showed that regulation of emotions using cognitive control strategies to downregulate emotional responses increased blood oxygenation level-dependent (BOLD) activity in the PFC and dACC, and decreased BOLD activity in the amygdala and the insula. This inverse relationship between frontal cortical and subcortical areas was predominantly observed in cognitive reappraisal tasks [15], [21], [22], and was directly related to the performance of emotion regulation [23].

In addition to this inverse relationship of the BOLD activity in the amygdala and the insula, PFC activity—particularly in the medial prefrontal cortex (mPFC)—has been implicated in the integration of emotional state information [24]. Additionally, Quirk and Beer suggested that the role of the mPFC in emotion regulation was to maintain regulation goals and transfer them to other cortical areas [25]. Urry and colleagues reported that mPFC BOLD activity increased, was sustained, or decreased according to the reappraisal goal of increasing, maintaining, or decreasing emotional responses, respectively [26]. A similar finding was reported by Ochsner and colleagues who showed differences in mPFC BOLD activity between the increase and decrease conditions [15].

These findings suggest that cognitive reappraisal goals may be represented and maintained in PFC during emotion regulation. It is likely that dynamic interactions between the control system and the appraisal system underlie the representation of reappraisal goals in PFC [27]. However, the dynamics of cortical activity representing goal information during cognitive reappraisal remain unclear, largely because of the limited temporal resolution of fMRI. Because the time scale of the dynamic interactions between the appraisal system and the control system is much smaller than the dynamics of the BOLD signal, faster methods, such as EEG or MEG, may be required to investigate the temporal dynamics of PFC activity. However, few EEG/MEG studies have shown neural correlates of cognitive reappraisal goals. Therefore, this study aims to locate the representation of reappraisal goals in the temporal patterns of EEG oscillations during a cognitive reappraisal task. We posit that such representation of cognitive reappraisal goals in EEG activity would provide a basis for developing an online neurofeedback paradigm for the enhancement of emotion regulation ability.

In particular, we focused on gamma oscillations in EEG in relation to cognitive reappraisal goals. Although little is known about the direct relationship between gamma oscillations and cognitive reappraisal, there have been substantial findings to lead us to investigate gamma oscillations. First, gamma oscillations in EEG are known to be highly correlated with BOLD activity [28], [29]. Therefore, it is likely that reappraisal goals represented in BOLD activity in PFC would be reflected in gamma oscillations. Second, the induced gamma activity has been used as an important tool to understand the neural mechanism underlying emotional processing [30]. A number of studies showed that emotional stimuli induced higher gamma power compared to neutral stimuli [31]–[34]. Martini and colleague has revealed that unpleasant stimuli increased gamma power in the frontal regions as well as large-scale gamma phase synchronization across frontal and temporal regions [35]. Popov and colleague have revealed the increased gamma power and local cross-frequency coupling of alpha and gamma oscillations in the mPFC during cognitive reappraisal to decrease emotions in response to unpleasant stimuli [36]. Third, gamma oscillations are known to be associated with cognitive processes that can be recruited during the manipulation of emotion regulation, such as attention, memory [37], [38] and emotion intelligence [39], [40]. For instance, Müller and colleagues reported that gamma power increased during selective attention to emotional stimuli compared to neutral ones [33]. Recent studies have suggested that gamma oscillations are implicated in emotional intelligence that assesses how well a person understands self-emotion or mindfulness that refers to non-judgmental awareness of internal and external emotions [39]–[43].

Given that these findings suggest a close relationship between gamma oscillations and emotional and cognitive processes, we investigated two characteristics of gamma oscillations—spectral power and large-scale phase synchronization—during cognitive regulation of emotion. Specifically, we tested the following hypotheses to address how gamma oscillations would be modulated during cognitive reappraisal. First, we hypothesized that frontal gamma power would vary with different reappraisal goals. While it was uncertain whether increasing/decreasing emotions would increase/decrease gamma power, frontal gamma power might be affected by reappraisal goal information in PFC activity and/or emotional and cognitive processing outcomes directed by these goals. Second, we hypothesized that large-scale gamma phase synchronization over the whole brain would increase during cognitive reappraisal. As large-scale phase synchronization in scalp EEG reflects the engagement of brain-wide neural networks to support cognitive control [35], [44], [45], we expected that gamma phase synchronization would reflect cognitive regulatory efforts, and thus increase during cognitive reappraisal to increase or decrease emotional responses.

## Materials and Methods

### Participants

Twenty healthy young adults (9 men, 11 women; mean age = 22.4±2.41 years) participated in the study. All participants were right-handed and had normal or corrected-to-normal vision without any self-reported neurological or neuropsychological disorders. The Institutional Review Board (IRB) of Korea University approved this study, and all participants provided written informed consent after the study procedure had been explained to them.

### Experimental procedure

#### Emotion regulation tasks and stimuli

We devised a cognitive reappraisal task with three different reappraisal goals: to decrease, maintain, or increase emotional responses [46]. In each epoch, participants were provided with a visual cue about a reappraisal goal before they responded to a stimulus. These visual cues were displayed on a screen and consisted of an up arrow, down arrow, and dash to indicate the increase, decrease, and maintain conditions respectively. In the increase condition, the participants were asked to amplify the intensity of their emotional response to a presented stimulus. In the maintain condition, the participants were asked to respond naturally to a stimulus by being aware of their feeling and maintaining it. In the decrease condition, the participants were asked to reduce the intensity of their emotional response. The cognitive reappraisal strategy the participants used was the self-focused strategy [15], in which the participants changed their level of self-relevance to a stimulus; in other words, they attempted to feel more or less involved in the event depicted in the presented stimulus.

Each participant was presented with 132 emotional pictures selected from an in-house affective picture set. The picture set consisted of an equal number of positive and negative pictures. The pictures were selected based on normative ratings in valence (1 to 7; unpleasant to pleasant) and arousal (1 to 7; calm to exciting), where the ratings were obtained from a separate group of 50 healthy participants who did not participate in this emotion regulation study. For our study, we selected a set of emotional stimuli based on these normative ratings in arousal and valence such that pictures with high arousal level and both positive and negative valence were selected. The average (± standard deviation [SD]) normative ratings in valence of the selected stimuli were 2.59±1.05 for negative and 5.29±1.19 for positive pictures. The normative ratings in valence were significantly different between selected positive and negative pictures (*p*<0.01). The average normative ratings in arousal of the selected stimuli were 4.60±1.42 for negative and 4.60±1.39 for positive pictures, respectively. The normative ratings in arousal of the selected stimuli were significantly higher than for neutral pictures (average: 3.65±1.35) contained in the in-house affective picture set (*p*<0.01). The participants never saw the same picture twice during the experiment.

#### Task procedure

**Figure 1:**
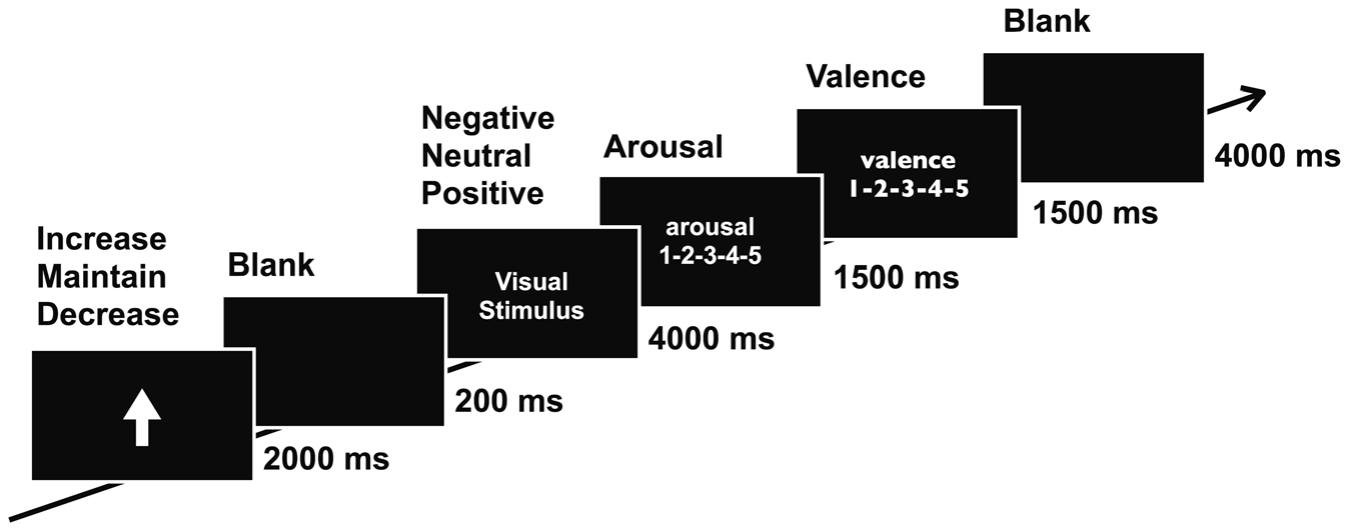
Timeline of the cognitive reappraisal task. Three regulatory instructions—increase, maintain, and decrease—were indicated by the up-arrow, dash, and down-arrow, respectively. After a 200 ms blank period, a 4000 ms visual emotional stimulus appeared for the cognitive reappraisal task. For the arousal and valence rating of the stimulus, two 5-point scale screens followed for 3000 ms each.

The task consisted of four blocks with a 5-min inter-block break. A total of 132 epochs containing all six conditions (3 reappraisal conditions×2 valence conditions) were randomly assigned to each block. A single epoch was composed of five consecutive periods (see Fig. 1). First, an epoch began with a blank screen presented for 1000 ms. Second, a visual cue appeared on the screen for 2000 ms to indicate a reappraisal goal. During this period, the participants acknowledged the meaning of the presented reappraisal goal and prepared to regulate their emotional response. Third, a 200-ms blank screen appeared again before stimulus onset. Fourth, an emotional picture was displayed on the screen for 4000 ms. During this period, the participants regulated their emotional response to the presented picture in accordance with the previously presented regulatory instruction cue. Picture presentation was pseudo-random, and no more than three pictures of the same valence condition were shown during consecutive epochs. Fifth, after the picture disappeared, assessment of the presented picture occurred. The participants were presented with a five-scale rating screen for 1500 ms to assess the valence level (1 = unpleasant to 5 = pleasant), followed by another rating screen for 1500 ms to assess the arousal level (1 = very low to 5 = very high). The participants rated the picture's five-scale valence and arousal levels by pressing one of five buttons on the computer keyboard.

#### EEG recording

Scalp EEG was recorded using 13 wet electrodes and the Grass Model 12A5 amplifier (Grass-Telefactor; An Astro-Med, Inc., West Warwick, Rhode Island, USA). The electrode locations (F3/z/4, C3/z/4, P3/z/4, O1/2, and T5/6) were determined according to the International 10–20 system. The EEG signal recorded from each electrode was sampled at 512 Hz, referenced to the right earlobe (A2), grounded using an electrode placed on the forehead (AFz), and re-referenced to the average activity of both earlobe electrodes. The vertical electrooculogram (vEOG) was recorded 1 cm below the right eye. Over the recording session, the impedance of each electrode was maintained below 5 KΩ. The EEG signal was digitized and then filtered using both a band-pass filter (0.1 to 100 Hz) to eliminate high-frequency noise and a notch filter at 60 Hz to attenuate power-line noise. To avoid the influence of edge effects arising from signal filtering and wavelet transform, the filtered EEG signal was first segmented into epochs of 6000 ms, starting 1000 ms before stimulus onset to 5000 ms after stimulus onset, passed through a series of signal processing steps with this extra data length, and then truncated back to the exact epoch length of 200 ms before to 4000 ms after stimulus onset. For the elimination of epochs contaminated by artifacts, such as eye movements, we performed independent component analysis (ICA) followed by visually inspecting EEG signals simultaneously with the vEOG. This noise reduction process removed an average of 40.4±25.7 contaminated epochs per participant, resulting in an average of 157.6±25.7 total epochs per participant.

#### Gamma band activity analysis

Since this study investigated how gamma band activity (GBA) was modulated during cognitive reappraisal tasks, we analyzed two primary aspects of GBA: spectral power and large-scale gamma phase synchronization. First, time-varying spectral power was estimated in the time-frequency domain by convoluting the EEG signal with the complex Morlet wavelet (see Equation 1) [47].
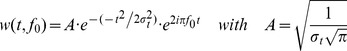
(1)The transform using the complex Morlet wavelet characterized higher frequency components with a fine temporal resolution and lower frequency components with a more precise frequency resolution [47]. For example, with the constant ratio of seven, the complex Morlet wavelet transform yielded the following resolutions in the time-frequency (TF) plane: SDs = 5 Hz, 31.8 ms with a 35-Hz wavelet; SDs = 7.86 Hz, 20.3 ms with a 55-Hz wavelet. This property provided an advantage to analyzing relatively high-frequency GBA with finer time resolutions. For each epoch, time-varying power in a given frequency band was calculated using the absolute value of the convolution of the EEG signal with the wavelet. Power values were log-transformed and normalized by subtracting the mean power value in the baseline period and then dividing by the standard deviation of the power value in the baseline period. The baseline period was the 200-ms period during the blank screen before stimulus onset. The gamma power analyzed in this study was the average of the power values in the gamma band ranging from 35 to 55 Hz.

Second, phase synchronization between a pair of EEG signals recorded from two different electrodes was estimated using the phase-locking value (PLV) [48], [49]. Electroencephalogram signals were filtered using a zero phase-lag finite impulse response (FIR) band-pass filter with a 2-Hz bandwidth, a central frequency (f_0_) ranging from 35 to 55 Hz, and a 1-Hz frequency step [48], [50]. The filtered signals were then transformed using the complex Morlet wavelet (with parameters identical to above) to obtain estimates of instantaneous phases. An instantaneous PLV for time instant (*t*) and for the frequency (*f*) between a pair of EEG signals at channels *n* and *m* was calculated as (see Equation 2),
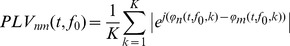
(2)where *K* is the number of trials and *n*, *m*, *t*, *f*, and *k* are instantaneous phases for the *k*th epoch. The instantaneous PLV ranged from zero (i.e., no phase coupling) to one (i.e., perfect phase coupling). To obtain a normal distribution, the calculated PLV was Fisher's *z*-transformed [51].

#### Statistical analyses

To identify what caused significant differences in frontal gamma power and when it occurred, we performed a three-way repeated-measures analysis of variance (RMANOVA) with the factors of reappraisal goals (i.e., decrease, maintain, increase), valence types (i.e., negative, positive), and frontal laterality (i.e., left, midline, right) on the gamma power data. This analysis was repeatedly applied to each 20-ms time window that moved from stimulus onset to stimulus offset (i.e., 4000 ms after onset). We also conducted post-hoc analyses using one-paired *t*-tests to assess the differences within the 20 ms within each factor. All analyses were conducted using Bonferroni adjustments of the *p*-value. We also investigated significant differences in the PLVs across reappraisal goals in two steps. In the first step, we performed a randomization test with 200 surrogate data sets to assess whether the instantaneous PLVs for each EEG pair exhibited a significant difference [48], [50]. If the original instantaneous PLV was significantly higher than the distribution of the surrogate instantaneous PLVs (*p*<0.01), we determined that the instantaneous PLV indicated significant phase synchronization. Otherwise, we did not include that instantaneous PLV in the next step. Following the randomization test, we averaged the significant instantaneous PLVs across the frequencies (21 frequencies over 35 to 55 Hz) for each time instant. In the second step, we tested the difference in PLVs between two different reappraisal goals (i.e., increase vs. maintain and maintain vs. decrease) for a given EEG channel pair. To investigate overall PLV patterns over time and to avoid possible misinterpretations by analyzing the PLV in an overly short time interval, we divided an epoch into 11 segments, including one baseline segment with a 200-ms duration and 10 post-stimulus segments each with a 400-ms duration. We then calculated the average time of the frequency-averaged instantaneous PLVs in each segment. Using these time-frequency-averaged PLVs from all the epochs, we repeatedly performed one-tail paired *t*-tests to determine differences between two reappraisal goals in each segment.

## Results

### Topographic map of gamma power associated with the three reappraisal goals

**Figure 2:**
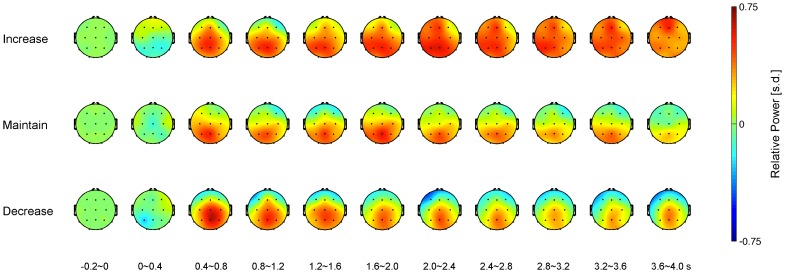
Time-varying topographic maps of gamma power in three different cognitive reappraisal conditions. Gamma power in each of the 13 EEG channels (see small black dots) was subtracted from the mean of the baseline power and divided into the standard deviation of the baseline power, resulting in the relative power. Bottom labels represent the time windows based on visual stimulus onset (increase, top; maintain, mid; decrease, bottom).

For an overall view of time-varying gamma activity, we built time-varying topographic maps of gamma power per cognitive reappraisal goal, as shown in Figure 2. We observed the maximum gamma power over the parietal-occipital regions irrespective of regulatory goals, which is consistent with our previous study [52]. Gamma power over the whole brain appeared to be higher and more sustained in the increase condition than in the other conditions. Additionally, gamma power in the frontal regions in the decrease condition was reduced compared to the other conditions.

### Spectral power and statistical analyses

**Figure 3:**
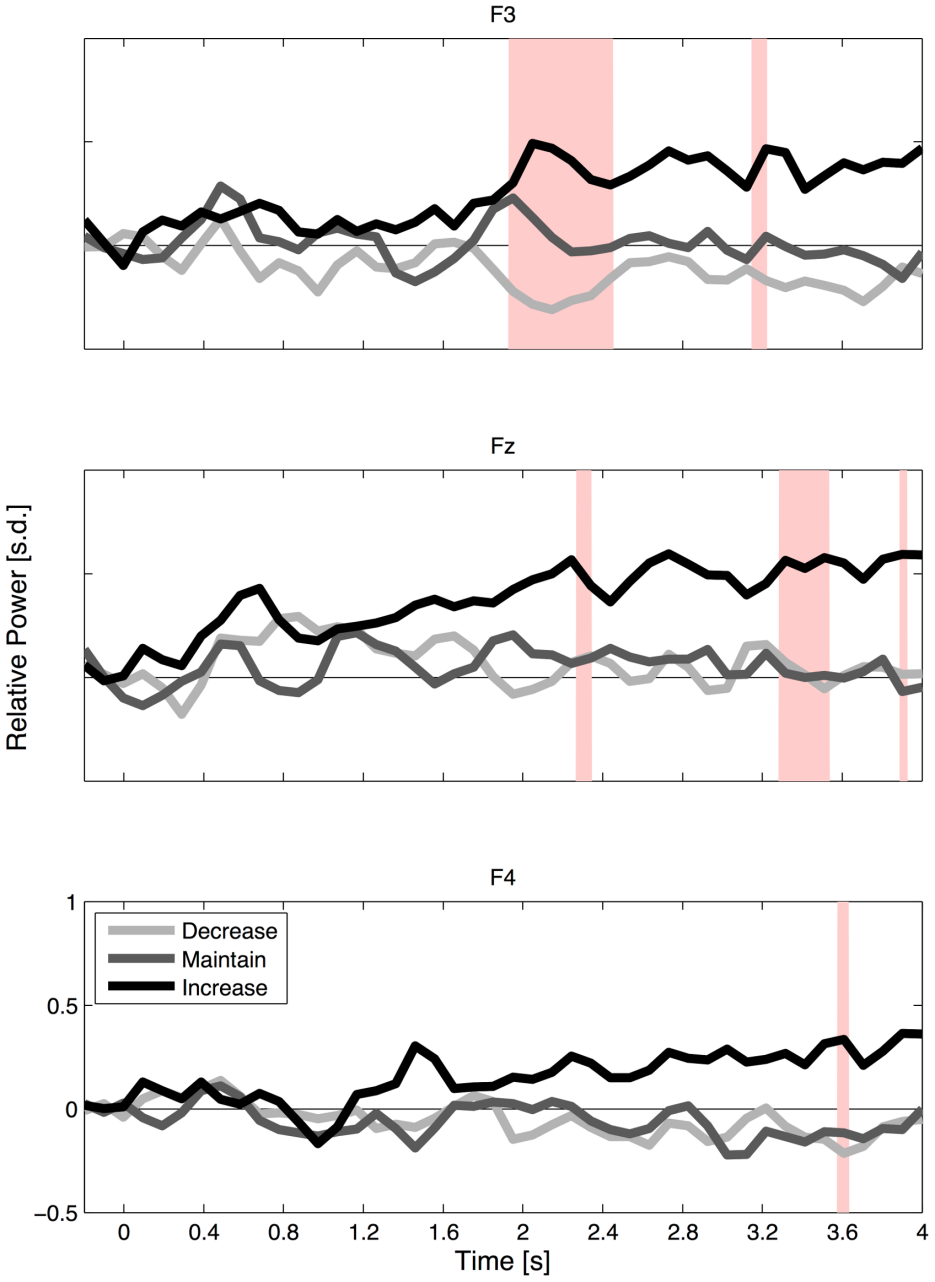
Temporal changes in frontal gamma power. Time-varying gamma activity at the left (F3, top), mid (Fz, mid), and right (F4, bottom) frontal regions during the cognitive reappraisal tasks. Lines indicate the decrease (light gray), maintain (dark gray), and increase (black) conditions. The light red areas in each panel indicate the significant difference durations (*p*<0.01).

Three-way RMANOVA (3 reappraisal goals × 2 valence levels × 3 frontal laterality positions) were conducted in the 20-ms time windows without overlap during the presentation of stimuli to identify whether and when significant differences for the within-factors occurred. The analysis revealed that stimuli began to induce significant main effects of reappraisal goals and frontal laterality after the 2000 ms after stimulus onset, while there was no significant main effect of valence levels and no interactions within all combinations across factors for all frontal channels and time windows (*p*<0.01). Figure 3 depicts the temporal patterns of the frontal gamma power for the different reappraisal goals. The analysis of temporal patterns showed that frontal gamma power began to diverge according to the cognitive reappraisal goals at approximately 2000 ms after stimulus onset. Additionally, it is noteworthy that compared to the baseline level (marked by the horizontal lines at the zero level in Fig. 3), gamma power at the left frontal region (F3) increased, decreased, or did not change. We assigned two separate time segments, including the windows with significant main effects, to a mid-period (1926 to 2453 ms) and a late-period (3293 to 3625 ms), and performed a post-hoc analysis with Bonferroni adjustment in these segments.

**Figure 4:**
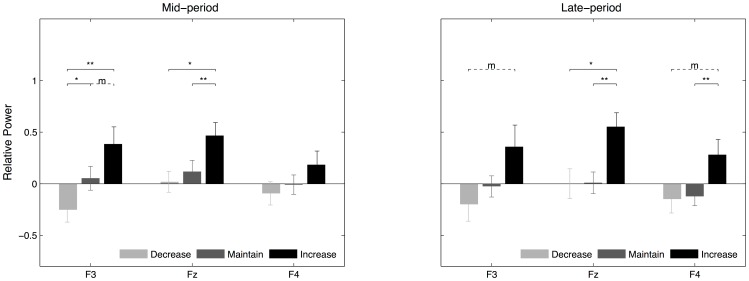
Statistical results in the mid and late interval. Comparison of frontal gamma power across three cognitive reappraisal goals (decrease, light black; maintain, dark gray; increase, black) in the mid (left) and late (right) interval. The error bars indicate standard error of the mean (SEM).

In the post-hoc analysis, we compared gamma power in the mid-period and late-period across reappraisal goals at each frontal channel (see Fig. 4). In the mid-period, the left frontal activity (F3) showed more differences across conditions compared to the mid (Fz) and right (F4) frontal activities. At the F3 channel, gamma power exhibited a monotonic pattern (i.e., decrease<maintain<increase) with a significant difference between the decrease and maintain conditions (paired *t*-test: *t*(19) = 2.53; *p*<0.05) and a marginally significant difference between the maintain and increase conditions, *t*(19) = 2.19, *p* = 0.065. At the Fz channel, gamma power in the increase condition was significantly higher than in the maintain condition, *t*(19) = 3.37, *p*<0.001, and decrease condition, *t*(19) = 2.92, *p*<0.05, but there was no difference between the maintain and decrease conditions, *t*(19) = 1.00, *p* = 0.166. At the F4 channel, no significant difference was observed between any pairing of reappraisal conditions (*p*>0.05). In the late-period, gamma power in the increase condition was higher than that in the decrease condition at all three channels but this difference reached significance only at Fz: F3, *t*(19) = 2.26, *p* = 0.063; Fz, *t*(19) = 2.85, *p* = 0.015, and F4, *t*(19) = 2.21, *p*<0.060. In addition, gamma power in the increase condition was significantly higher than in the maintain condition at Fz, *t*(19) = 3.91, *p*<0.01, and F4, *t*(19) = 3.09, *p*<0.01. In summary, only the mid-period gamma power in the left frontal region (F3) showed a monotonically increasing pattern consistent with the reappraisal goals (i.e., decrease<maintain<increase).

The RMANOVA revealed that neither the main effect of valence type nor the interaction between reappraisal goals reached level of significance. We performed further statistical analyses to clarify whether negative and positive stimuli modulated similar or different patterns of gamma power during the mid-period and late-period using one-paired *t*-tests. For negative stimuli, the gamma power for both the increase and maintain conditions was larger than for the decrease condition at the F3 channel during the mid-period; this difference was marginally significant for the maintain condition and significant for the increase condition (decrease vs. maintain, *t*(19) = 2.15, *p* = 0.066; decrease vs. increase *t*(19) = 2.52, *p*<0.05). At the Fz channel, the gamma power for the increase condition was significantly higher than for the decrease, *t*(19) = 2.69, *p*<0.01, and maintain conditions, *t*(19) = 2.17, *p*<0.05, during the mid-period. During the late-period, more pronounced gamma power for the increase condition compared to the other conditions was obtained at the Fz channel with a significant difference (*p*s<0.01), and at the F4 channel with a marginally significant difference (*p*s<0.065), while the statistical differences across reappraisal goals disappeared at the F3 channel (*p*s>0.1). The positive stimuli elicited smaller differences in gamma power across the reappraisal goals than the negative stimuli, although both induced a similar pattern of gamma activity. Again, gamma power for the increase condition was higher than that for the other conditions at the F3 channel during the mid-period (increase vs. maintain *t*(19) = 2.17, *p* = 0.063; increase vs. decrease *t*(19) = 2.69, *p*<0.05). There were no significant differences across reappraisal goals during the late-period (*p*s>0.07). In sum, the negative stimuli generated more enhanced differences in gamma power across reappraisal goals than the positive stimuli.

### Gamma phase synchrony

**Figure 5:**
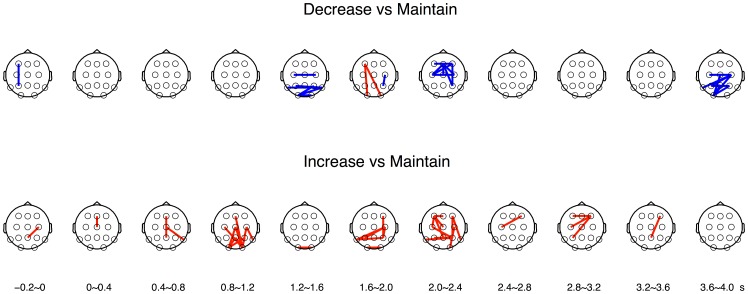
Differences of degree in PLV pairs between cognitive reappraisal conditions. The top panel shows the comparison between the decrease and maintain conditions (red: maintain>decrease; blue: maintain<decrease). The bottom panel depicts the comparison of the degree of PLV in all possible pairs between the increase and maintain conditions (red: increase>maintain; blue: increase<maintain). Significance level is *p*<0.01. Bottom labels represent the time windows based on visual stimulus onset.

We also tested how gamma phase synchronization across the brain varied across reappraisal goals. From the statistical analysis of PLV differences across different reappraisal goals in each time segment (paired *t*-tests, *p*<0.01; see Methods and Materials), we constructed a series of topographic maps for the phase-synchronized EEG channel pairs that exhibited significant differences in PLVs between a pair of reappraisal conditions (see Fig. 5). We observed that relatively more EEG pairs showed higher PLVs in the decrease condition than in the maintain condition, predominantly from 1200 to 2400 ms after stimulus onset. Across all time segments after stimulus onset, 28 pairs showed higher PLVs in the decrease condition, whereas only two pairs showed higher PLVs in the maintain condition. We also found relatively more EEG pairs showing higher PLVs in the increase condition than in the maintain condition, predominantly from 800 to 1200 ms, and 2000 to 3200 ms. Across all time segments, 37 pairs showed higher PLVs with the increase condition, whereas no pair showed higher PLVs with the maintain condition. Between the increase and decrease conditions, few EEG pairs showed significant differences in PLVs.

## Discussion

In this study, we investigated how gamma oscillations in human EEG varied with cognitive reappraisal goals to decrease, maintain, or increase emotional responses to external visual stimuli. We analyzed two aspects of gamma oscillations: spectral power and large-scale phase synchronization. We found that frontal gamma power was modulated by reappraisal goals whereby it increased, sustained, or decreased with the regulatory goals of increasing, maintaining, or decreasing emotional responses, respectively. This linear modulation of frontal gamma power with reappraisal goals appeared in the left frontal region during the period of 1926 to 2453 ms after viewing an emotional picture. Regarding large-scale phase synchronization, there were more phase-synchronized pairs of gamma oscillations over the whole brain for the decrease and increase goal conditions compared to the maintain goal condition.

We also observed maximum gamma power over the parietal region regardless of cognitive reappraisal goals. Parietal neural activity related to emotional processing has been largely studied using the LPP. Previous studies have reported that variations in LPP amplitudes were modulated by emotional stimuli [8], [10], [11] and emotion regulation tasks [9], [13], [14], [53]. Overall, the LPP amplitude was enhanced by emotional stimuli relative to neutral stimuli, and downregulated by cognitive reappraisal. These phenomena were found predominantly in centro-parietal regions and were associated with the degree of attention and the reappraisal of emotional stimuli [13], [23], [54]. However, there is a lack of evidence for the association of neural mechanisms underlying LPP modulation with the control system of emotion regulation implemented in the frontal cortex. In line with this perspective, Hajcak and colleagues recently addressed the importance of the frontal cortex for emotion regulation, and the necessity of investigating the relationship between gamma oscillations and individual differences in emotional response and regulation [13].

In contrast to the parietal LPP, a close relationship between the frontal cortex and emotion regulation processes has been revealed based on the results from many fMRI studies [15], [26], [55]–[57]. Ochsner and colleagues showed that both up and downregulation of negative emotion activated the PFC and the ACC [15]. Additionally, they revealed that upregulation of negative emotion activated the left-hemisphere, whereas downregulation of negative emotion activated both hemispheres. Using both positive and negative stimuli, Kim and Hamann reported that PFC activity was increased by the regulatory condition and that positive upregulation involved left PFC regions, whereas negative downregulation involved right PFC regions [57]. Furthermore, Urry and colleagues showed that the activity in Brodmann area (BA) 10, a region of the mPFC, was linearly correlated with regulatory goals (i.e., decrease<maintain<increase) [26]. These results suggest that mPFC activity might be associated with regulatory goals for cognitive emotion regulation.

In general, the mPFC has been implicated in a wide range of cognitive tasks, such as mentalizing, problem solving, explicit processing of internal information, emotion regulation, and emotional task execution [24], [25], [58]–[60]. According to the gateway hypothesis proposed by Burgess and colleagues, the mPFC is engaged in the goal-directed coordination of stimulus-independent thought (SIT) and stimulus-oriented thought (SOT) when predetermined actions fail to achieve a goal [58]. They also suggested that the function of the mPFC is “metacognition” (i.e., referring to one's own thoughts in a consciously controlled and goal-directed mode) and evaluation, monitoring, or manipulation of internally generated information [58]. The mPFC has also been implicated in cognitive emotion regulation. Olsson and Ochsner reported that the mPFC was involved in the integration of information about internal body states and in the categorization of emotional states [24]. Quirk and Beer suggested that the role of the mPFC in emotion regulation is maintaining the goal of downregulating emotion and transferring this information to the orbitofrontal cortex (OFC), which then effects the suppression of amygdala activity [25]. These previous studies suggest that the mPFC integrates emotional state information and manipulate a regulatory goal during cognitive reappraisal.

The timing of frontal gamma power modulation during cognitive reappraisal suggests that this modulation reflects a closed-loop system including feedback from the appraisal system. We observed frontal gamma power modulation approximately 2000 ms after stimulus onset. If frontal gamma power solely represented the top-down control process, it should have appeared much earlier than 2000 ms (i.e., top-down emotion regulation effectively reduced the LPP that typically appears 300 to 800 ms) [13]. Therefore, it is likely that our observation of frontal gamma power modulation reflects the maintenance of reappraisal goals by the PFC that is supported by closed-loop information transfer between the control and appraisal systems.

It is also noteworthy that frontal gamma power modulation appeared only during the middle interval, from 1926 to 2453 ms after stimulus onset. Notably, both the significant difference of frontal gamma power across reappraisal goals and the increase of gamma phase synchronization for regulatory efforts occurred simultaneously in this period. After this period, such a difference of frontal gamma power became insignificant and gamma phase synchronization began to decrease (see Fig. 5). This observation suggests that frontal gamma power might reflect cortical representation of emotional states modulated by cognitive reappraisal processes involving regulatory efforts indicated by increases in gamma phase synchronization.

Our results showed the left frontal lateralization of gamma power during cognitive appraisal. Several neuroimaging studies has suggested that left frontal regions was more involved in emotional processing than right frontal regions by showing that differences in BOLD activity modulated by pleasant and unpleasant stimuli were bigger in the left DLPFC than in the right DLPFC [61], [62]. Considering the positive correlations between gamma oscillations and BOLD activity [28], [29], our results may suggest that left frontal gamma oscillations reflect the cortical representation of emotional states in the left DLPFC modulated by the reappraisal goals.

Overall, we also found strong gamma phase synchronization across EEG channels during cognitive reappraisal that may be an indicator of increased functional connectivity during reappraisal trials. In general, large-scale phase synchronization between brain oscillations represents communication between distant neural assemblies [63]. Furthermore, increased gamma phase synchronization has been observed in many cognitive tasks, including visual perception [47], [49], [50], learning [64], emotional processing [35], and emotion regulation [36]. These studies indicate that conscious cognitive efforts, such as cognitive reappraisal, elicit increases in large-scale gamma phase synchronization in the brain. Our findings are also in line with previous fMRI studies showing an increased functional coupling between cortical and subcortical regions during conscious reappraisal of emotions [21], [27], [65].

Furthermore, we observed that gamma phase synchronization appeared earlier in the increase condition than in the decrease condition (see Fig. 5). We surmise that this temporal difference might be related to the level of regulatory difficulty. Previous studies reported that decreasing emotion was more difficult than increasing emotion [15], [57], owing to the difficulty of reversing emotional reactivity. Therefore, it is likely that faster gamma phase synchronization in the increase condition compared to the decrease condition was due to the relatively easy and fast execution of emotion regulation.

Despite these robust effects of frontal gamma activity associated with cognitive reappraisal, there are still several limitations to our study that should be addressed by future work. First, since we mainly focused on the temporal variations of brain oscillations modulated by different stimulus types and reappraisal goals, the number of EEG channels was insufficient to represent variations of local gamma power and global gamma phase synchronization. In fact, this limitation kept us from applying EEG source localization methods to investigate gamma oscillation sources in the frontal cortex and other regions. Additionally, it would be interesting to examine functional connectivity between localized sources that are related to emotional regulatory processes. Second, we excluded neutral stimuli in this study to ensure the presence of emotional responses in all cognitive reappraisal conditions. The use of neutral stimuli might be confusing in our study, as it would have been difficult for participants to manipulate their emotional responses to neutral stimuli without evoked emotions. Thus, neutral stimuli might result in unexpected cognitive processes irrelevant to emotion regulation when participants attempt to reappraise their responses. However, the exclusion of neutral stimuli in our study raises the issue of confirmation of emotional responses modulated by the pictures, because the participants' responses between emotional and neutral stimuli might have provided support for the validity of emotional responses elicited by the different picture set. To partially resolve this issue, we selected a set of pictures from the in-house picture database with high-arousal normative ratings. This database was standardized in terms of ratings in arousal and valence, and we carefully collected pictures such that their arousal ratings were clearly higher than for the neutral pictures (*p*<0.01). In addition, a recent study by Ahn and colleagues on emotional memory effects, using the same stimuli as in our study, showed that these emotional pictures were recalled better than neutral pictures one week after presentation, supporting the notion that this picture set elicited emotional responses in participants [46]. However, to completely confirm the induction of emotional experiences by our stimulus set, it would be necessary to use neutral stimuli together with emotional ones in future studies.

In addition, previous studies have reported stimulus-preceding negativity (SPN), an ERP component, which was generally elicited before the onset of stimulus during the emotion regulation [14], [66]. According to these studies, the SPN amplitude during the preparation period was modulated by emotion regulation conditions. Therefore, it would be interesting to investigate in future studies how neural oscillations (particularly gamma oscillations) are modulated by cognitive reappraisal during this preparation period.

Identifying neural correlates of emotion regulation processes may aid in the development of new methods for the treatment of emotion-related psychiatric disorders. For example, a cognitive training program that provides neurofeedback may assist patients in regulating their emotional responses in real-time. In fact, recent studies have demonstrated the feasibility of providing online feedback for emotion regulation by presenting the amygdala activity from BOLD signals in real time [67]–[69]. However, current fMRI-based methods possess critical weaknesses, including a significant time delay from neural activity to presentation, and environmental restrictions during the magnetic resonance (MR) scanning (e.g., the subject must lie inside the scanner). In contrast, an EEG-based method can provide greater mobility and a finer time resolution that is critical for online feedback. Recent advances in the development of dry and mobile EEG sensors would add practicality to the use of EEG in neurofeedback applications. However, little effort has been made to utilize EEG signals for neurofeedback of emotion regulation, predominantly because of the lack of evidence for EEG correlates of regulation goals. However, our results indicating the representation of regulatory goals in frontal gamma activity of EEG may provide a new opportunity to develop effective neurofeedback training systems for emotion regulation.
